# Medium- and Long-Term Effectiveness of Custom Insoles for Cavus Foot: A Surface Electromyography Study

**DOI:** 10.3390/jfmk10040461

**Published:** 2025-11-25

**Authors:** Sara García-Bautista, Antonio Gómez-Bernal, Javier Alfaro-Santafé, Jose Luis Perez-Lasierra

**Affiliations:** 1 Faculty of Medicine, University of Zaragoza, 50009 Zaragoza, Spain; 837717@unizar.es; 2 Podoactiva Biomechanical Unit, Research & Development Department, Parque Tecnológico Walqa, Ctra. N330a Km 566, 22197 Cuarte, Spain; antoniogomez@podoactiva.com (A.G.-B.); javieralfaro@podoactiva.com (J.A.-S.); 3 Department of Podiatry, Faculty of Health Sciences, Manresa University, 08243 Manresa, Spain; 4 Facultad de Ciencias de la Salud, Universidad San Jorge, Villanueva de Gállego, 50830 Zaragoza, Spain

**Keywords:** cavus foot, insoles, foot orthoses, electromyography, muscle contraction

## Abstract

**Background:** Cavus foot, defined by an increased medial longitudinal arch and often forefoot plantarflexion, alters biomechanics and increases peak plantar pressures, raising the risk of musculoskeletal disorders such as metatarsalgia, Achilles tendinopathy, and gait instability. Custom foot orthoses are the preferred conservative treatment, offering plantar support, pressure redistribution, and reduction in compensatory muscle activity. This study evaluated the medium- and long-term effectiveness of custom orthoses in 71 patients with cavus feet using surface electromyography (sEMG) and the same shoes. **Methods:** Muscle activity of the peroneus longus, peroneus brevis, and gastrocnemius was recorded during treadmill gait after one and four months of orthotic use. **Results:** Significant reductions in muscles were observed, especially after four months, confirming greater long-term effectiveness. No residual benefits were found when participants walked without orthoses. **Conclusions:** These findings support the clinical value of insoles in reducing the compensatory muscle activity in cavus feet and emphasize the importance of investigating their long-term role in biomechanics and potential pathology risk reduction.

## 1. Introduction

In the general population, various foot types can be observed, among which cavus foot stands out with a prevalence of 8.55%; however, this prevalence can reach nearly 50% in stages 1 and 2, particularly in women [1,2]. This foot type is characterized by an increased medial longitudinal arch and, in many cases, forefoot plantarflexion. The etiology of cavus foot is multifactorial, including congenital, neuromuscular, acquired, and idiopathic causes [1,3,4,5,6,7,8,9]. Due to its morphological characteristics, cavus foot exhibits a reduced plantar contact area, leading to higher peak pressure distribution. Additionally, its gait biomechanics differ from those of other foot types, showing decreased external tibial rotation and increased rearfoot dorsiflexion. In the coronal and transverse planes, inversion and abduction of the rearfoot are observed, respectively. Regarding the forefoot, plantarflexion occurs in the sagittal plane, valgus alignment in the coronal plane, and adduction in the transverse plane [4,7,8,9].

These alterations may increase the risk of developing pathologies involving muscular, osseous, ligamentous, tendinous, and cartilaginous structures. The most frequent conditions include gait instability; ankle pain; painful plantar calluses; decreased metatarsal sensitivity; claw toe deformities; metatarsalgia; and, in some cases, knee and hip pain [3,4,5].

Due to its morphological characteristics, cavus foot imposes increased demands on the musculotendinous system, requiring compensatory muscle activity to preserve foot stability and functionality. This may lead to overload and imbalance that, over time, can result in pathologies such as tendinopathies, plantar fasciitis, among others [9,10,11]. These cavus-foot-related pathologies are characterized by alterations in normal muscle activation patterns. Such alterations may involve excessive activation of specific muscle groups, such as the gastrocnemius or peroneal muscles, as a compensatory response to structural instability. Conversely, insufficient activation may arise from fatigue or neuromuscular inhibition. These changes in muscle electrical activity not only reflect the presence of pathology but also provide insight into its persistence or progression. Surface electromyography enables the identification and quantification of these activation differences, making it a valuable tool for both diagnosis and the development of more effective treatment strategies [12,13,14,15,16,17,18].

In the scientific literature, various treatment approaches have been described for this foot type and its potentially associated pathologies. In most cases, conservative management is considered the treatment of choice, with custom-made insoles being the most widely used intervention among healthcare professionals [10,11,19,20,21,22,23]. Custom insoles provide structural support and stabilization to the foot, contributing to a more homogeneous redistribution of plantar pressures [3,4,5]. This treatment is also effective for most conditions associated with cavus foot and is frequently integrated with other multidisciplinary therapeutic approaches [1,3,4,5]. On the other hand, surgical treatment aimed at structurally modifying the foot to reduce the height of the plantar arch is indicated only in a limited number of cases [1,3,5,7,9,10,22].

Research suggests that plantar supports molded to the plantar surface of the foot and covered with cushioning promotes an optimal redistribution of pressures, reducing peak loads in both the metatarsal and rearfoot areas [11,17,20,23]. Additional components can be incorporated into the plantar support to enhance stability, prevent further structural deformation of the foot, and contribute to reducing the electromyographic activity of the stabilizing muscles [9,10,11,19,20,21], which is frequently altered. Objectively quantifying the effects of treatment can often be challenging; therefore, employing instruments that evaluate muscle electrical activity, such as electromyography, may be of great value. These instruments serve two main purposes: first, to identify distinct patterns of electrical activity that may facilitate the detection or diagnosis of human disorders, and second, to determine whether the applied treatment is effective [12,13,14].

Electromyography, in addition to its other applications, has long been employed to assess neuromuscular responses across different activities. Furthermore, it provides valuable quantitative information on neurophysiological processes, allowing the examination of central and peripheral nervous system control [14,15,24]. Its effectiveness has been demonstrated across various fields, particularly in neurophysiology, through the assessment of neural activation patterns, muscle activity, and human movement strategies [24]. Its role has also been acknowledged in pathophysiology, both as a diagnostic tool for neuromuscular disorders and as a means of evaluating the rehabilitation of patients with these conditions and the effectiveness of their treatments [24,25].

Several studies have demonstrated that muscle electrical activity decreases with the use of insoles in patients with cavus feet, leading to improvements in both health and quality of life in the short and medium terms [20,25,26,27]; however, others have reported no significant changes over similar follow-up periods [23,28]. Moreover, no study to date has assessed the effectiveness of custom insoles in the long term, which underlines the need for the present investigation. In this context, the objective of this study was to evaluate the medium- and long-term effectiveness of custom insole treatment through surface electromyography in patients with cavus feet.

## 2. Materials and Methods

### 2.1. Study Design and Participant Recruitment

A quasi-experimental study was designed with the objective of assessing the medium- and long-term effects of the proposed treatment following its application. Recruitment was performed through verbal invitations to patients attending the podiatry clinic at Podoactiva Sagasta (Zaragoza, Spain). Those who met the inclusion criteria were provided with detailed information about the study and invited to participate. The inclusion criteria were as follows:

• Sex and age: Male and female participants over 18 years old.
• Condition: Diagnosis of cavus feet, confirmed through pressure platform analysis and visual inspection of elevated plantar arches during a biomechanical assessment [29].
• Health: No presence of conditions that could influence the structures under evaluation in the study, such as peripheral and central nervous system neuropathies, polymyositis, poliomyelitis, muscular dystrophy, herniated disc, myasthenia gravis, Guillain-Barré syndrome, Charcot-Marie-Tooth disease, amyotrophic lateral sclerosis, or multiple sclerosis.
• Footwear: Consistent use of the same participant’s personal casual walking footwear during all evaluation sessions.
• Treatment adherence: Wearing the treatment for more than 5 days/week and for at least 8 h/day.

The study received prior approval from the Ethics Committee of Aragón (CEICA), under reference number (PI23/371). It was conducted in compliance with the principles of the Declaration of Helsinki for research involving human subjects [30].

### 2.2. Assessment of Treatment Effectiveness Through Muscle Electrical Activity During Gait

Treatment effectiveness during gait was assessed at both the medium-term (>1 month) and long-term (>4 months) following initiation of the intervention. To this end, a protocol was implemented to evaluate the electromyographic activity of various lower limb muscle groups while participants walked at a constant speed on a treadmill.

The evaluation protocol for muscle electrical activity consisted of walking on a treadmill at a speed of 3 km/h, with no incline, for a duration of 1 min [25]. Muscle electrical activity was assessed using the mDurance surface electromyography system (Shimmer3 EMG hardware (Shimmer Research, Dublin, Ireland) integrated with a mobile application and a cloud-based analysis platform (mDurance Solutions S.L., Granada, Spain)). This validated instrument [16,17] enables reliable evaluation of muscle activation during dynamic muscle contractions in real time, as evidenced by several research studies [16,17]. For data acquisition, round Ag/AgCl electrodes with solid gel were used [13,14]. Muscle activation was recorded from the peroneus longus (P Long) and peroneus brevis (P Brevis), as well as from the medial gastrocnemius (Med G) and lateral gastrocnemius (Lat G) of both lower limbs.

Regarding electrode placement, two electrodes were positioned on the Med G at its greatest prominence, aligned with the leg; the reference electrode was placed on the lateral malleolus. For the Lat G, two electrodes were placed at one third of the distance between the head of the fibula and the heel. For the P Long muscle, the electrodes were positioned at one quarter of the distance from the highest point between the head of the fibula and the lateral malleolus. Finally, the electrodes for the P Brevis muscle were placed at one quarter of the distance from the lowest point between these same landmarks [13,14,18].

In total, each participant underwent two assessment sessions, scheduled as follows:

• First assessment: After 36.7 ± 9.7 days of treatment use, muscle electrical activity was assessed under two conditions: First, footwear without insoles and second, footwear with insoles.
• Second assessment: After 162.2 ± 42.3 days of treatment use, muscle electrical activity was assessed under the same two conditions: First, footwear without insoles and second, footwear with insoles.

During all assessment sessions, each participant wore the same footwear. The primary outcome variable used to evaluate muscle electrical activity was the root mean square value (in microvolts) of the signal, following the Lux protocol [31].

### 2.3. Assessment of Sociodemographic and Health Factors

During the initial visit, participants completed a general questionnaire that recorded data such as date of birth, body weight, height, and cavus foot type. Additional information was obtained regarding medical conditions that could influence foot function, previous injuries within the past 12 months, current injuries, and the specific foot region in which discomfort was reported.

Cavus foot was classified into three levels of severity (Grades 1, 2, and 3), following the classification proposed by Cavanagh and Rodgers. Grade 1 cavus foot was defined as a mildly increased medial longitudinal arch, characterized by an arched plantar support with an isthmus width less than one-third of that of a normal footprint. Grade 2 cavus foot was identified when this narrowing resulted in a hiatus while preserving its anterior and posterior extensions. Grade 3 cavus foot was defined as the absence of an isthmus imprint [32].

### 2.4. Statistical Analysis

Qualitative variables were presented as absolute frequencies and percentages, while quantitative variables were first assessed using the Shapiro–Wilk test to verify normal distribution. When data met the normality assumption, they were described using the mean and standard deviation; otherwise, the median and interquartile ranges (Q1–Q3) were employed. To compare two conditions within the same subjects, a paired *t*-test was applied in parametric cases, including effect size estimation with Cohen’s d. For non-parametric cases, the Wilcoxon signed-rank test was used, and effect size was reported using the r coefficient. When comparisons were made between three independent groups, one-way ANOVA was applied for normally distributed data, while the Kruskal–Wallis test was used when normality was not met. For categorical variables, the chi-square test was employed. To evaluate changes before and after the intervention, paired tests were applied: for parametric data, the paired *t*-test was used, reporting the mean, standard deviation, t statistic, and Cohen’s d; for non-parametric data, the Wilcoxon signed-rank test was used, reporting the median, interquartile range (Q1–Q3), W statistic, and effect size through Wilcoxon’s r. In all analyses, the statistical significance level was set at α = 0.05 and calculations were performed using R statistical software (version 4.3.3).

## 3. Results

Of the 105 initial participants who completed the first assessment (conducted one month after the initial visit), 92 also completed the second assessment, as 13 were unable to attend due to personal reasons. Among the 92 participants who completed all assessments, 71 attended wearing the same footwear in all sessions; therefore, the remaining 21 participants were excluded from the data analysis. All 71 participants adhered to the treatment protocol, and none wore the insoles less than the minimum duration required (5 days/week and 8 h/day), as verified through self-reported logs completed by the participants.

Of the 71 study participants, 39.44% presented Grade 1 cavus foot, 32.39% Grade 2 cavus foot, and 28.17% Grade 3 cavus foot (Table 1). Participants with Grade 1 cavus foot had, on average, a higher body weight compared to the other groups. Regarding injuries within the past 12 months, no differences were observed except for knee pain, and hip pain which were more prevalent among participants with Grade 2 cavus foot, with a prevalence of 26.1% and 17.4%, respectively. As for current injuries, the most prevalent were calf/soleus muscle overload and Achilles tendinopathy, along with mechanical metatarsalgia, both more common in participants with Grade 3 cavus foot (45.0%). A higher frequency of pain was observed in the inner heel and medial ankle, the foot arch, and the forefoot region, particularly among participants with Grade 3 cavus foot. The results are shown in Table 1.

### 3.1. Medium Term Effectiveness of the Treatment

**Figure 1:**
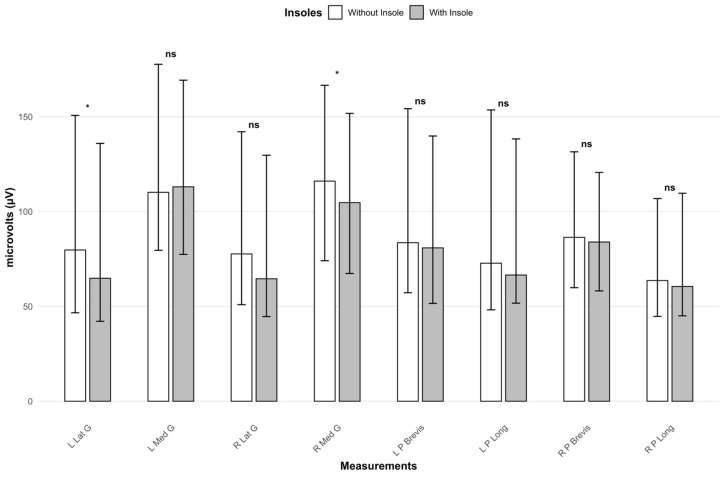
Representation of all electrical activity measurements from the first evaluation across two conditions (footwear with insoles, footwear without insoles). *: Significance level < 0.05. ns: Significance level > 0.05. L Lat G: Left lateral gastrocnemius. L Med G: Left medial gastrocnemius. R Lat G: Right lateral gastrocnemius. R Med G: Right medial gastrocnemius. L P Brevis: Left peroneus brevis. L P Long: Left peroneus longus. R P Brevis: Right peroneus brevis. R P Long: Right peroneus longus.

Muscle electrical activity data were analyzed under the two evaluated conditions (footwear with insoles and footwear without insoles) during the first assessment (Table 2; Figure 1). When using the treatment, significant differences were observed compared to not using it in the left Lat G (64.85 vs. 79.76; *p* = 0.021; effect size = 0.246) and in the right Med G (104.74 vs. 116.08; *p* = 0.024; effect size = 0.241), with lower activation in both cases when using the insoles compared to not using them. No statistically significant differences were found in the remaining variables analyzed (Table 2; Figure 1).

### 3.2. Long Term Effectiveness of the Treatment

**Figure 2:**
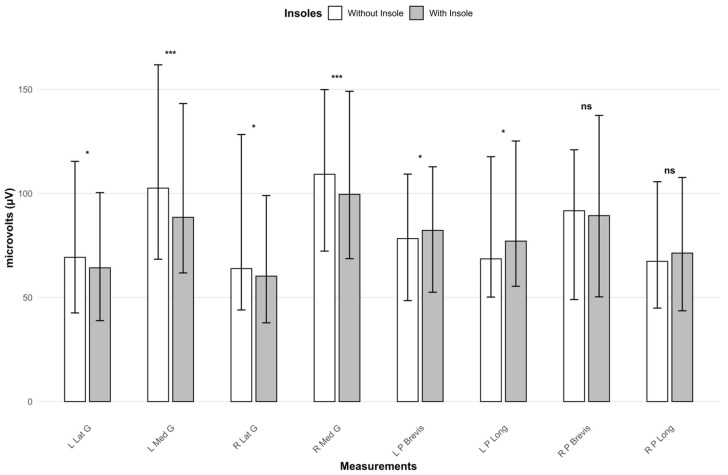
Representation of electrical activity in the second evaluation (footwear with insoles, footwear without insoles). *: Significance level < 0.05. ***: Significance level < 0.001. ns: Significance level > 0.05. L Lat G: Left lateral gastrocnemius. L Med G: Left medial gastrocnemius. R Lat G: Right lateral gastrocnemius. R Med G: Right medial gastrocnemius. L P Brevis: Left peroneus brevis. L P Long: Left peroneus longus. R P Brevis: Right peroneus brevis. R P Long: Right peroneus longus.

Regarding the results of the second assessment, muscle electrical activity data were analyzed under the two evaluated conditions (footwear with insoles and footwear without insoles) (Table 3; Figure 2). When using the treatment, significant differences were observed compared to not using it in the left Lat G (64.27 vs. 69.30; *p* = 0.013; effect size = 0.295), left Med G (88.54 vs. 102.56; *p* < 0.001; effect size = 0.415), right Lat G (60.28 vs. 63.91; *p* = 0.013; effect size = 0.296), and right Med G (99.57 vs. 109.22; *p* < 0.001; effect size = 0.407), showing a reduction in muscle electrical activity with the use of insoles.

On the other hand, significant differences were observed in the left P Brevis (82.24 vs. 78.31; *p* = 0.021; effect size = 0.273) and left P Long (77.09 vs. 68.57; *p* = 0.044; effect size = 0.239), with muscle activation being greater when using insoles compared to not using them. No statistically significant differences were found in the remaining variables analyzed.

### 3.3. Electrical Activity Evolution Without Treatment

**Figure 3:**
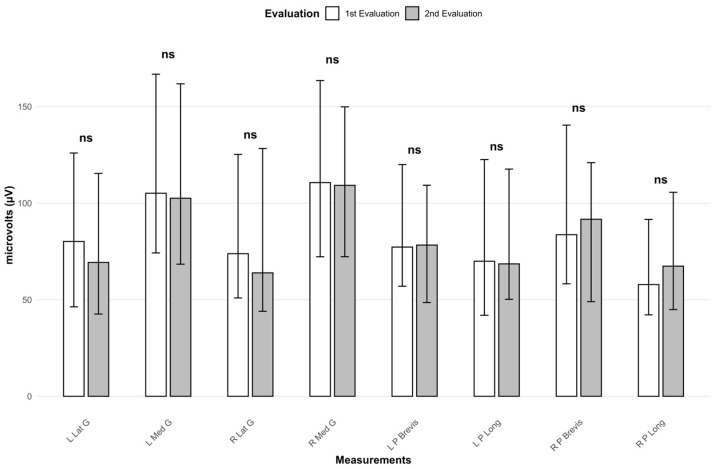
Representation of electrical activity in the first and second evaluation (footwear without insoles). ns: Significance level > 0.05. L Lat G: Left lateral gastrocnemius. L Med G: Left medial gastrocnemius. R Lat G: Right lateral gastrocnemius. R Med G: Right medial gastrocnemius. L P Brevis: Left peroneus brevis. L P Long: Left peroneus longus. R P Brevis: Right peroneus brevis. R P Long: Right peroneus longus.

Additionally, the persistence of treatment effects was evaluated by comparing muscle electrical activity between the two assessments sessions when participants were not wearing the treatment. As shown, no significant differences were observed in any of the variables analyzed between the two evaluations when participants were not wearing insoles in their footwear (Table 4; Figure 3).

## 4. Discussion

The results of the study demonstrated a significant reduction in muscle electrical activity in patients with cavus foot when using custom insoles, highlighting the effectiveness of the treatment at the medium-term after one month of use, but especially at the long term when the treatment had been applied for more than four months. When comparing the medium- and long-term evaluations, the reduction in muscle activity was already evident after one month of use but became more pronounced after four months in most of the muscle groups analyzed. This progressive improvement suggests a cumulative neuromuscular adaptation to the orthosis, reinforcing the importance of prolonged and continued treatment to achieve the full therapeutic effect.

Furthermore, the findings indicate that the treatment is only effective while being used, showing no residual efficacy when insoles are not worn, even after a prolonged period of prior use. No significant differences were observed between the two assessments when the insoles were not worn. This effect indicates that the neuromuscular benefits of custom insoles are not retained once the orthosis is removed, meaning that the therapeutic effect depends on continued use. From a clinical perspective, this finding underlines the importance of consistent daily wear to achieve and maintain functional improvements. It also highlights the need for clinicians to emphasize adherence when prescribing orthotic treatment for cavus foot, as intermittent use may limit therapeutic outcomes. For patients, this reinforces the practical message that the beneficial biomechanics provided by the insoles are not permanent, and therefore require regular use to support symptom reduction and gait efficiency.

The main results of the study are consistent with those reported by similar studies, which have shown a reduction in electrical activity in different lower limb muscles when using custom insoles in participants with cavus foot [20,25,26,27]. Nevertheless, it is important to note the lack of statistical significance in the results at medium-term regarding the activation of the P Long and P Brevis muscles, which may be somewhat controversial when considering previous similar studies [20]. However, the absence of statistical differences in our study may have been influenced by the heterogeneity of the sample, as participants presented with different pathologies and received personalized and individualized, thus diverse, treatments for their conditions. This suggests that future studies could explore this aspect with a more homogeneous sample. Additionally, the peroneal muscles are part of the lateral muscle chain, which contributes both to stabilization and mobility of the lower limb, being involved in abduction, external rotation, and dynamic postural control. This multifunctionality could explain the more variable behavior of the peroneal muscles compared to other muscles analyzed, since they are part of this lateral muscle chain. Due to these multiple functions, their neuromuscular activity often shows greater variability in activation patterns and intermuscular coordination. In contrast, the posterior musculature generally has a more specific and homogeneous role, mainly related to extension and propulsion during gait, which results in a more stable and predictable behavior in the recordings analyzed. The study conducted by Moisan et al. has reported a possible reduction in peroneal muscle electrical activity in patients who used specific customization elements in their insoles, such as lateral controls or bars [20]. The results of the study showed that the incorporation of lateral controls into the insole led to a reduction in muscle electrical activity in the P Long and Lat G, probably due to the greater lateral stability provided by the additional support. However, other muscles, such as the tibialis anterior, gluteus medius, and vastus lateralis, showed an increase in electrical activity, particularly at the beginning of the stance phase [20]. It is important to note that these results may introduce biases in interpretation, since in other phases of the gait cycle, or when using insoles without a lateral control, tibialis anterior activity tends to decrease. Therefore, the influence of the insole may vary significantly depending on the phase of analysis within the gait cycle [20].

Interestingly, the increased activation of peroneus brevis and longus in the long-term evaluation suggests that orthoses may redistribute the stabilization demand from the posterior to the lateral muscles, reflecting a functional adjustment toward improved lateral control and load distribution in cavus foot. This pattern reflects a functional adjustment aimed at enhancing lateral control and improving load distribution in the cavus foot, where elevated medial arch height and hindfoot varus typically shift the center of pressure laterally. By promoting a more balanced loading pattern, the insoles may reduce excessive reliance on the posterior chain and facilitate a more physiologically efficient gait strategy [33]. However, this effect was only observed after long-term application and in one extremity, so it should be interpreted with caution pending further evidence from future studies.

Other similar studies have reported a significant increase in the electrical activity of the tibialis anterior, Med G, quadriceps, hamstrings, and erector spinae muscles when using insole modifications [23]; however, it should be noted that their objective was to observe changes produced by adding a heel lift, and therefore, the treatment they employed was not fully customized, which differs substantially from the intervention used in our study. Another study with 15 participants with cavus foot evaluated the effectiveness of custom insoles in reducing electrical activity in different muscle groups (Med G and Lat G, gluteus medius, vastus lateralis and medialis, biceps femoris, P Long, and tibialis anterior), but their results did not show significant differences [28]. Nevertheless, it should be taken into account that the insoles were used for only one month, which likely conditioned the effectiveness of the treatment. This becomes more relevant when considering the results of our study, which demonstrate the clear effectiveness of the treatment in the long-term (after at least 4 months) and the difference in effectiveness compared to just one month of use.

Additionally, a clinically relevant finding emerged from the baseline assessment. Participants with grade 2 cavus foot reported significantly higher hip pain (17.4%, *p* = 0.014). This may indicate a proximal biomechanical compensation mechanism because cavus foot increases lateral loading and forefoot stiffness, which alters lower limb alignment and increases demands on proximal stabilizers such as gluteus medius and hip rotators. Therefore, the presence of hip pain in grade 2 participants may reflect an overload of proximal muscles due to compensatory strategies [34].

Regarding the assessment protocol, a controlled walking speed of 3 km/h was selected to ensure methodological consistency across participants and to minimize variability in muscle activation that may arise from changes in gait velocity. Walking speed is known to influence lower limb kinematics and EMG patterns, and increasing velocity artificially can induce compensatory strategies that confound the interpretation of neuromuscular responses. By standardizing speed at a low, safe, and reproducible level, we ensured that the differences observed in EMG activity were attributable to the insole condition rather than to speed-dependent biomechanical changes. This approach is consistent with previous research on cavus foot, where participants also walked at a controlled treadmill speed of 3 km/h [25].

The present study has several strengths that reinforce the internal and external validity of its findings. First, it is worth highlighting the use of a large sample size compared to similar studies, which typically included only 10–15 participants, thereby limiting their external validity. Second, the application of the treatment and long-term follow-up provided a more comprehensive understanding of the actual effectiveness of custom insoles, improving upon the evidence presented in previous studies where follow-up lasted only one month and involved very small samples. Finally, the methodology employed was robust, as a validated electromyography system was used, whose reliability has been confirmed in previous research [16,17], which ensures accuracy in the measurement of muscle electrical activity. Homogeneity in footwear use was also considered, as all participants wore the same footwear in all evaluations, thereby reducing variability and increasing the consistency of the results. However, our study has some limitations. First, although the treatment used was the same custom insoles, their very nature of being tailored to each individual makes them inherently different, which increases heterogeneity; nevertheless, despite this variability, consistent neuromuscular patterns were still observable across participants. Second, although the effect of footwear was controlled by including only participants who used the same shoes in all assessments, possible design variations among them (e.g., different heel drop, cushioning) must be considered, as these could have influenced the results. Additionally, future studies could include muscles like the tibialis anterior to provide a more comprehensive view of arch stabilization.

## 5. Conclusions

The use of custom insoles in patients with cavus feet leads to a significant reduction in muscle electrical activity, with effects becoming evident after one month of use and being more pronounced after four months of use. However, these benefits are only present while the insoles are worn, as no residual efficacy is observed once their use is discontinued, indicating that the neuromuscular effects do not persist after removal, highlighting the clinical importance of consistent daily use. These findings emphasize the importance of continued research on the long-term biomechanical effects of custom foot orthoses and their role in preventing related pathologies.

## Figures and Tables

**Table 1:** Descriptive data of all participants and comparisons across cavus foot grades.

	Overall	Cavus Grade 1	Cavus Grade 2	Cavus Grade 3	*p* Value
Number of participants, %	100 (71)	39.44 (28)	32.39 (23)	28.17 (20)	
Age, years	46.0 (28.0, 59.0)	42.0 (23.0, 60.0)	46.0 (25.0, 59.0)	47.0 (35.0, 58.0)	0.5 ^1^
Weight, kg	71.61 (14.26)	77.96 (15.48)	70.57 (10.66)	63.90 (12.40)	0.002 ^2^
Height, cm	171.28 (10.81)	172.21 (10.21)	172.30 (11.11)	168.80 (11.40)	0.5 ^2^
Injuries in the last year, %					
None	49.3 (35)	53.6 (15)	43.5 (10)	50.0 (10)	0.8 ^2^
Plantar fasciitis	4.2 (3)	7.1 (2)	0.0 (0)	5.0 (1)	0.6 ^3^
Knee pain	19.7 (14)	21.4 (6)	26.1 (6)	10.0 (2)	0.5 ^3^
Hip pain	5.6 (4)	0.0 (0)	17.4 (4)	0.0 (0)	0.014 ^3^
Cervical and lumbar pain	4.2 (3)	3.6 (1)	4.3 (1)	5.0 (1)	>0.9 ^3^
Calf/soleus overload and AT	5.6 (4)	3.6 (1)	8.7 (2)	5.0 (1)	0.8 ^3^
Peroneal muscle overload	2.8 (2)	3.6 (1)	0.0 (0)	5.0 (1)	0.7 ^3^
Mechanical metatarsalgia	8.5 (6)	3.6 (1)	8.7 (2)	15.0 (3)	0.4 ^3^
Posterior tibial tendinopathy	2.8 (2)	3.6 (1)	0.0 (0)	5.0 (1)	0.7 ^3^
Other injuries	14.1 (10)	10.7 (3)	17.4 (4)	15.0 (3)	0.8 ^3^
Current injuries, %					
None	100 (71)				
Plantar fasciitis	14.1 (10)	14.3 (4)	8.7 (2)	20.0 (4)	0.6 ^2^
Knee pain	14.1 (10)	17.9 (5)	17.4 (4)	5.0 (1)	0.4 ^2^
Hip pain	2.8 (2)	3.6 (1)	4.3 (1)	0.0 (0)	>0.9 ^2^
Cervical and lumbar pain	2.8 (2)	3.6 (1)	4.3 (1)	0.0 (0)	>0.9 ^2^
Calf/soleus overload and AT	38.0 (27)	35.7 (10)	34.8 (8)	45.0 (9)	0.7 ^3^
Peroneal muscle overload	11.3 (8)	17.9 (5)	8.7 (2)	5.0 (1)	0.4 ^2^
Mechanical metatarsalgia	26.8 (19)	17.9 (5)	21.7 (5)	45.0 (9)	0.090 ^3^
Posterior tibial tendinopathy	9.9 (7)	7.1 (2)	13.0 (3)	10.0 (2)	0.9 ^2^
Other injuries	16.9 (12)	17.9 (5)	21.7 (5)	10.0 (2)	0.6 ^2^
Pain and location, %					
None	18.3 (13)	17.9 (5)	30.4 (7)	5.0 (1)	0.11 ^2^
Forefoot and toes	32.4 (23)	25.0 (7)	30.4 (7)	45.0 (9)	0.3 ^3^
Foot arch	22.5 (16)	14.3 (4)	21.7 (5)	35.0 (7)	0.2 ^2^
Foot lateral	9.9 (7)	17.9 (5)	8.7 (2)	0.0 (0)	0.14 ^2^
Inner heel and inner ankle	26.8 (19)	25.0 (7)	13.0 (3)	45.0 (9)	0.059 ^3^
Outer heel and outer ankle	5.6 (4)	7.1 (2)	4.3 (1)	5.0 (1)	>0.9 ^2^
Back of the ankle	15.5 (11)	14.3 (4)	8.7 (2)	25.0 (5)	0.4 ^2^
Plantar surface	4.2 (3)	7.1 (2)	4.3 (1)	0.0 (0)	0.8 ^2^
Top of the foot	4.2 (3)	7.1 (2)	4.3 (1)	0.0 (0)	0.8 ^2^

^1^ Kruskal–Wallis rank sum test, ^2^ Fisher’s exact test, ^3^ Pearson’s Chi-squared test, AT: Achilles tendinopathy. The number in parentheses refers to the number of participants in qualitative variables and standard deviation or interquartile range in quantitative variables.

**Table 2:** All electrical activity measurements from the first evaluation across two conditions (footwear with insoles, footwear without insoles).

	With Insoles	Without Insoles	*p*-Value	Eff Size
L Lat G	64.85 (42.14, 135.98)	79.76 (46.65, 150.76)	0.021	0.246
L Med G	113.08 (77.45, 169.31)	110.17 (79.59, 177.69)	0.470	0.077
R Lat G	64.58 (44.63, 129.74)	77.68 (50.95, 142.12)	0.090	0.181
R Med G	104.74 (67.36, 151.80)	116.08 (74.10, 166.63)	0.024	0.241
L P Brevis	80.88 (51.60, 139.88)	83.59 (57.22, 154.29)	0.125	0.164
L P Long	66.57 (51.71, 138.33)	72.80 (48.18, 153.61)	0.195	0.138
R P Brevis	83.96 (58.18, 120.65)	86.41 (59.87, 131.54)	0.465	0.078
R P Long	60.51 (45.02, 109.68)	63.65 (44.73, 106.89)	0.858	0.019

All muscle variables were analyzed using the non-parametric Wilcoxon signed-rank test. Values are reported as median and interquartile ranges (Q1–Q3). Effect size corresponds to r. L Lat G: Left lateral gastrocnemius. L Med G: Left medial gastrocnemius. R Lat G: Right lateral gastrocnemius. R Med G: Right medial gastrocnemius. L P Brevis: Left peroneus brevis. L P Long: Left peroneus longus. R P Brevis: Right peroneus brevis. R P Long: Right peroneus longus.

**Table 3:** Electrical activity in the second evaluation (footwear with insoles, footwear without insoles).

	With Insoles	Without Insoles	*p*-Value	Eff Size
L Lat G	64.27 (38.88, 100.4)	69.30 (42.59, 115.43)	0.013	0.295
L Med G	88.54 (61.81, 143.22)	102.56 (68.41, 161.81)	<0.001	0.415
R Lat G	60.28 (37.84, 99.01)	63.91 (44.02, 128.32)	0.013	0.296
R Med G	99.57 (68.69, 149.08)	109.22 (72.29, 149.89)	<0.001	0.407
L P Brevis	82.24 (52.53, 112.81)	78.31 (48.52, 109.29)	0.021	0.273
L P Long	77.09 (55.39, 125.2)	68.57 (50.2, 117.66)	0.044	0.239
R P Brevis	89.35 (50.33, 137.50)	91.69 (49.03, 120.99)	0.320	0.118
R P Long	71.34 (43.63, 107.68)	67.39 (44.91, 105.65)	0.152	0.170

All muscle variables were analyzed using the non-parametric Wilcoxon signed-rank test. Values are reported as median and interquartile ranges (Q1–Q3). Effect size corresponds to r. L Lat G: Left lateral gastrocnemius. L Med G: Left medial gastrocnemius. R Lat G: Right lateral gastrocnemius. R Med G: Right medial gastrocnemius. L P Brevis: Left peroneus brevis. L P Long: Left peroneus longus. R P Brevis: Right peroneus brevis. R P Long: Right peroneus longus.

**Table 4:** Electrical activity in the first and second evaluation (footwear without insoles).

	1st Evaluation	2nd Evaluation	*p*-Value	Eff Size
L Lat G	80.18 (46.31, 125.99)	69.30 (42.59, 115.43)	0.154	0.169
L Med G	105.16 (74.25, 166.79)	102.56 (68.41, 161.81)	0.848	0.023
R Lat G	73.84 (50.93, 125.24)	63.91 (44.02, 128.32)	0.331	0.116
R Med G	110.64 (72.26, 163.5)	109.22 (72.29, 149.89)	0.512	0.078
L P Brevis	77.27 (57, 120.02)	78.31 (48.52, 109.29)	0.229	0.143
L P Long	69.93 (41.95, 122.58)	68.57 (50.2, 117.66)	0.686	0.048
R P Brevis	83.66 (58.26, 140.42)	91.69 (49.03, 120.99)	0.600	0.063
R P Long	57.85 (42.17, 91.58)	67.39 (44.91, 105.65)	0.893	0.016

All muscle variables were analyzed using the non-parametric Wilcoxon signed-rank test. Values are reported as median and interquartile ranges (Q1–Q3). Effect size corresponds to r. L Lat G: Left lateral gastrocnemius. L Med G: Left medial gastrocnemius. R Lat G: Right lateral gastrocnemius. R Med G: Right medial gastrocnemius. L P Brevis: Left peroneus brevis. L P Long: Left peroneus longus. R P Brevis: Right peroneus brevis. R P Long: Right peroneus longus.

## Data Availability

The data presented in this study are available on request from the corresponding author due to ethical reasons.

## Abbreviations

The following abbreviations are used in this manuscript:

sEMG	Surface electromyography
CEICA	Ethics Committee of Aragón
L Lat G	Left lateral gastrocnemius
L Med G	Left medial gastrocnemius
R Lat G	Right lateral gastrocnemius
R Med G	Right medial gastrocnemius
L P Brevis	Left peroneus brevis
L P Long	Left peroneus longus
R P Brevis	Right peroneus brevis
R P Long	Right peroneus longus
ANOVA	Analysis of variance
AT	Achilles tendinopathy
